# Supplementary material for: Patterns of Intron Gain and Loss in Fungi
Source: PLoS Biol. 2004 Nov 30;2(12):e422. doi: 10.1371/journal.pbio.0020422 (PMC532390; doi:10.1371/journal.pbio.0020422)
Supplement: Table S1 — Also available at http://genes.mit.edu/NielsenEtAl/. (4.3 MB ZIP). [file pbio.0020422.st001.zip › NielsenEtAl/html/574.html]

AN5957.1.NCU04292.1.MG00942.1.FG04570.1


```
 CLUSTAL W (1.82) Multiple Sequence Alignments - Introns Inserted


Sequence 1: MG00942.1	419 aa
Sequence 2: FG04570.1	407 aa
Sequence 3: NCU04292.1	434 aa
Sequence 4: AN5957.1	400 aa
Alignment Length: 471 aa
Number Identitical Residues: 154 aa
Alignment Score (without introns) 8871


MG00942.1 	----MAPPAAITPVTPGTPEPSLNKGGPDTNLTAAAIGHKMS----------QASEYQEL
NCU04292.1	MVSHSEPP--------------------KEDLSAVALEHTIDEANALINGTSDVPKIQEL
FG04570.1 	----MAPSAVET---------------QDVNLTAAAIHKKEVAQ-------PTTTTQAPL
AN5957.1  	---------------------------------MGSIG----------------NTLAEL
          	                                   ::                      *

MG00942.1 	DASKLIYNLTT-SPRALPDYDTAAAGSETVCTDHMVTANWHAATGWSAPTIKPYGPLSLM
NCU04292.1	DASKLVVTLADPNARQVPDEATANAASETICTDHMVTVSYNVESGWGVPELKPYGPISLM
FG04570.1 	DASKLTYTYTQ-NPRSVPDEANAHSGDETIATDHMVLAAWKQSTGWAAPELKPYGPLSLM
AN5957.1  	DASVVKITRST-ELRHVPLPGSLEELSHSYCTDHMVTARWTAAGGWETPEVKPFQNLSIP
          	*** :  . :  . * :*   .    ..: .***** . :    ** .* :**:  :*: 

MG00942.1 	PTASVLHYATECFEGLKAYRGYDGKLRLFRPDRNANRMLMSSVRIALPSFPPEALEKLLI
NCU04292.1	PTASVLHYATECFEGLKVFRGFDGKLRLFRPNLNAKRLLMSTLRIALPGFDPVEVEKLIA
FG04570.1 	PTASVLHYATECFEGLKVYRGYDGKLRIFRPDRNAARLRMSSTRISLPSFEPSEIEKLMI
AN5957.1  	PTASCLHYATECFEGMKVYRGFDGKLRLFRPDLNGERLSNSAVRASLPSFRFQELKTLIA
          	**** **********:*.:**:*****:***: *. *:  *: * :**.*    ::.*: 

MG00942.1 	ALMAVDGPK~WLPRERPGTLLYLRPTMIGTQAQLGVQAPNQAMLFITASYFP----TMDS
NCU04292.1	KLVAVDGAK~WLPKDRPGSFLYIRPAVIGTQPQLGVQAPKQALLFITVSFMP----RMDS
FG04570.1 	SLLSVDGTK~WLPADRAGSFLYIRPTVIGTAPQLGVSAPKEALLYIIVTFMP----RMDA
AN5957.1  	KLMQIDGLR1WLPKDQPGRFLYLRPTLIGSGTQLGVQAPAEALLFIIAVPWPDPATRLKA
          	 *: :** : *** ::.* :**:**::**: .****.** :*:*:* .   *..:: :.:

MG00942.1 	PAG---GMRLHTSPEDMVRAWVGGFGYAKVGANYGPSLLATKEAKSRGFHQILWLYGPEG
NCU04292.1	PAG---GMRLHTNPEDMIRAWVGGFGYAKVGANYGPSLLATNEARARGFHQILWLYGKEA
FG04570.1 	PPG---GMRLHTSPDDMVRAWVGGFGYAKVGANYGPSLLATADARTRGFHQILWLYGSQG
AN5957.1  	TPGEALGLKLLTSAPDTIRAWPGGFGYAKLGANYGPSLAAHGKAQAQGFDQVLWLFGEDR
          	..*.: *::* *.. * :*** *******:******** *  .*:::**.*:***:* : 

MG00942.1 	YCTEAGASNFFVLMKNKQ--TGRLEIVTAPLDDRVILGGITRMSVLELARSRLG------
NCU04292.1	YCTEAGASNFFIVWRAKESEGGKLQLLTAPLDDKLILDGVTRRSVLELAKARLA------
FG04570.1 	ECTEAGASNFFVVWKRK---DGKKEIITAPLDDKLILDGVTRRSCLELIRERLG------
AN5957.1  	QVTEAGASNFFIVWENAQ--TGKRELVTAPLENQLILPGVTRRSVLELARSRLNQAVGDL
          	  *********:: .  .   *: :::****::::** *:** * *** : ** .: .. 

MG00942.1 	DEVDVVERRYTIDEVFEAYAEGRIVESFASGTA0YFVCPISLIHHRGKEVSIPMGEDGQG
NCU04292.1	GEVEVVERRYTIDEVIEADKEGRIVEAFAAGTA0FFIAPVSQIHHRGTDVHIPLG-ENDS
FG04570.1 	DEYEITERKYTISEVMEADAEGRLLESFAAGTA0YFICPISQIHHRGKDINIPMGAEGEA
AN5957.1  	EAVEVVEKTFTIWDVEAAWKEGRVVEAFVCGTA0FFITPVKLIRNG--AVDIQLLKPGQT
          	   ::.*: :** :*  *  ***::*:*..*** :*: *:. *::    : * :   .: 

MG00942.1 	GKYTLKLKQWLKDIMYGN---EQHEWGVVVPEE~Q-------------------
NCU04292.1	GVYTTKIKSWMKDIMYGN---EQHEWGVVINEE2DEAVAGQKRKREQNEEAKRE
FG04570.1 	GEVTSKVKGWLGDIMYGR---TEHPWGVVIPEK~KQ------------------
AN5957.1  	AGYAAQIKSWLEAVMYGKDGAENHEWSYIIENE~SEK-----------------
          	.  : ::* *:  :***...: :* *. :: :: ..
```
